# Negligible risk of inducing resistance in *Mycobacterium tuberculosis* with single-dose rifampicin as post-exposure prophylaxis for leprosy

**DOI:** 10.1186/s40249-016-0140-y

**Published:** 2016-06-08

**Authors:** Liesbeth Mieras, Richard Anthony, Wim van Brakel, Martin W. Bratschi, Jacques van den Broek, Emmanuelle Cambau, Arielle Cavaliero, Christa Kasang, Geethal Perera, Lee Reichman, Jan Hendrik Richardus, Paul Saunderson, Peter Steinmann, Wing Wai Yew

**Affiliations:** Netherlands Leprosy Relief, P.O. Box 95005, 1090 HA Amsterdam, The Netherlands; Royal Tropical Institute (KIT), Amsterdam, The Netherlands; Swiss Tropical and Public Health Institute, Basel, Switzerland; Independent TB and Leprosy Consultant, Helmond, The Netherlands; French National Reference Center for Mycobacteria, Paris, France; Novartis Foundation, Basel, Switzerland; The German Leprosy and Tuberculosis Relief Association, Würzburg, Germany; Ministry of Health, Colombo, Sri Lanka; New Jersey Medical School Global Tuberculosis Institute, New Jersey, USA; Erasmus MC, Rotterdam, The Netherlands; American Leprosy Missions, Greenville, USA; Chinese University of Hong Kong, Hong Kong, China

**Keywords:** Post-exposure prophylaxis, Leprosy, Mycobacterium tuberculosis, Rifampicin resistance

## Abstract

Post-exposure prophylaxis (PEP) for leprosy is administered as one single dose of rifampicin (SDR) to the contacts of newly diagnosed leprosy patients. SDR reduces the risk of developing leprosy among contacts by around 60 % in the first 2–3 years after receiving SDR. In countries where SDR is currently being implemented under routine programme conditions in defined areas, questions were raised by health authorities and professional bodies about the possible risk of inducing rifampicin resistance among the *M. tuberculosis* strains circulating in these areas. This issue has not been addressed in scientific literature to date. To produce an authoritative consensus statement about the risk that SDR would induce rifampicin-resistant tuberculosis, a meeting was convened with tuberculosis (TB) and leprosy experts. The experts carefully reviewed and discussed the available evidence regarding the mechanisms and risk factors for the development of (multi) drug-resistance in *M. tuberculosis* with a view to the special situation of the use of SDR as PEP for leprosy. They concluded that SDR given to contacts of leprosy patients, in the absence of symptoms of active TB, poses a negligible risk of generating resistance in *M. tuberculosis* in individuals and at the population level. Thus, the benefits of SDR prophylaxis in reducing the risk of developing leprosy in contacts of new leprosy patients far outweigh the risks of generating drug resistance in *M. tuberculosis*.

**Electronic supplementary material:**

The online version of this article (doi:10.1186/s40249-016-0140-y) contains supplementary material, which is available to authorized users.

## Multilingual abstract

Please see Additional file 1 for translations of the abstract into the six official working languages of the United Nations.

## Introduction

In post-exposure prophylaxis (PEP) for leprosy, one single dose of rifampicin (SDR) (600 mg for adults and appropriately reduced doses for children) is administered to the contacts of newly diagnosed leprosy patients to reduce their risk of developing leprosy. These contacts comprise household members, neighbours and defined social contacts, and may include around 20 persons (with a range of 5 – 50) per leprosy patient. Robust evidence for the effectiveness of SDR as PEP for leprosy has been generated through two large field-level trials. The first was a non-randomised controlled trial in Indonesia [1] and the second a randomised double-blind placebo-controlled field trial in Bangladesh [2]. In both studies, SDR administered to contacts of leprosy patients provided close to 60 % protection against developing leprosy among contacts within the first 2–3 years after receiving SDR. Interestingly, the size of the effect was comparable to that of more prolonged dapsone prophylaxis, which was tested in the 1960s and 70s [3]. The effect was sustained for up to 10 years of follow-up. Sub-group analysis suggested that when SDR was given to subjects who had had Bacillus Calmette-Guérin (BCG) immunization in infancy, the protective effect reached 80 %. A new clinical trial designed to specifically test the protective effect of SDR plus a further dose of BCG in contacts, is currently under way [4].

The leprosy post-exposure prophylaxis (LPEP) project is now being implemented in selected areas of India, Indonesia, Myanmar, Nepal, Sri Lanka and Tanzania by the respective Ministries of Health in partnership with members of the International Federation of Anti-Leprosy Associations (ILEP) and scientific partners, under coordination and with support from the Novartis Foundation. The project aims to assess the feasibility of implementing SDR as PEP for leprosy under routine programme conditions and evaluate the impact of the intervention on leprosy incidence over time.

During the preparatory work for the LPEP project, questions and doubts were raised by health authorities and professional bodies about the possible risk of inducing rifampicin resistance among the *Mycobacterium tuberculosis* (*M. tuberculosis*) strains circulating in the areas were the LPEP project is implemented. The scientific literature has not specifically addressed this issue to date.

For this reason, an expert meeting was convened to assess the risk of drug resistance in *M. tuberculosis* by administering SDR to contacts of leprosy patients. This high level consultation involved experts in both leprosy and TB from a range of disciplines and had as its objective to produce an authoritative consensus statement about the risk that SDR would induce rifampicin-resistant TB.

## Review

### Mechanisms related to drug resistance in mycobacterium tuberculosis

The World Health Organization (WHO) estimates that there are 9 million TB patients globally with an annual mortality of 1.1 million cases (excluding cases with a combined TB/HIV infection). The number of patients with multidrug-resistant TB (MDR-TB) is estimated at 480 000, among whom 210 000 deaths occur every year [5].

The vast majority of TB patients can be cured with a standard 6-month treatment course of four antimicrobial drugs comprising of isoniazid, rifampicin, ethambutol and pyrazinamide in the first 2-month intensive phase, followed by isoniazid and rifampicin in the subsequent 4-month continuation phase. Correct dosing and regular drug intake are essential to ensure treatment success and reduce the risk of drug resistance. MDR-TB is caused by *M. tuberculosis* resistant to, at least, isoniazid and rifampicin, the two most powerful first-line anti-TB drugs. The primary cause of MDR-TB is irregular treatment but inappropriate or incorrect use of anti-TB drugs and use of poor quality medicines also contribute to drug resistance formation.

Drug resistance in *M. tuberculosis* is the result of the sequential accumulation of mutations in genetically isolated mycobacterial lineages [6]. As DNA replication is not perfect, any sufficiently large population of bacteria will randomly contain a small number of mutants with single nucleotide polymorphisms (SNPs) that may result in resistance to an antibiotic. Prolonged treatment of patients with a large enough population of bacteria (usually more than 10^8^ organisms) with a single anti-TB drug (e.g. rifampicin) will select the bacteria containing these SNPs [7]. Sustained antibiotic pressure provides an advantage for resistant mutants, as growth of the sensitive form is suppressed, and allows adaptation and genetic fixing of the resistant genotype [8, 9]. Sufficiently large bacterial populations for this genetic fixing to take place are found in patients with pulmonary TB, especially when cavities are present. In solid pulmonary lesions, extra pulmonary TB or in latent TB infections without pulmonary involvement, the number of organisms is small enough to make the generation of drug-resistant TB unlikely.

### Probability of the development of drug resistance in *Mycobacterium tuberculosis*

The probability of the emergence of MDR-TB depends on the potency and the number of different drugs used for treatment, and on the mycobacterial load [10]. With a potent and correctly administered multidrug regimen, the probability of drug resistance developing appears to be very low, even in the presence of high bacterial loads as found in pulmonary cavitary disease. However, a recent modeling study suggested that the probability of resistance development is several orders of magnitude higher than previous estimates [11]. Furthermore, there appears to be a possibility of mutagenesis from *M. tuberculosis* persisters (or stressed cells) transiently exposed to low concentrations of antimicrobial drugs [12]. Intermittent therapy with drugs thus portends a higher risk of developing resistance in *M. tuberculosis* than daily treatment, especially in HIV-infected subjects [13]. This is likely due to the regrowth of bacilli with different susceptibilities in the interval between two periods of chemotherapy [14].

In the original 14-day Early Bactericidal Activity (EBA) study, using either isoniazid, rifampicin or ofloxacin, it was found that drug resistance occurred in only 1 of 12 patients [15]. In early trials of isoniazid (INH) monotherapy in India, where half of the patients took INH alone for 12 months, it was shown that it took up to 12 months for INH resistance to appear [16]. Numerous subsequent EBA studies used isoniazid as a positive control and drug resistance was never encountered in tests done after less than 2 weeks of monotherapy [17]. This suggests that resistance development after a few doses of monotherapy is very rare, and thus highly unlikely to emerge after the administration of a single dose.

Acquired resistance to rifampicin in *M. tuberculosis* has also not been reported to be a frequent problem with rifamycin-based regimens for the treatment of latent TB infection (as only 2 out of 3 such trials had a few patients developing rifamycin-resistant TB), with the caveat that the currently available database is relatively small [18].

### Risk factors for inducing drug resistance in *M. tuberculosis* through SDR administration

Any use of antibiotics bears at least a minimal risk of resistance development [19]. Two risk factors are particularly important for the development of drug resistance in *M. tuberculosis*: (i) the prolonged and/or repeated exposure to a single antibiotic; and (ii) its administration to a person with a substantial load of *M. tuberculosis* bacilli. Neither risk factor is present in the context of SDR administration to contacts of leprosy patients. The intake of SDR is supervised and contacts are screened for classical symptoms of TB: cough (>2 weeks), night sweats, unexplained fever or weight loss.

Excluding contacts with any of the classical TB symptoms will reduce the risk of SDR being given to people with clinical TB and thus individuals harbouring a significant number of bacilli. Administration of SDR to an individual with sub-clinical TB disease or a latent TB infection bears negligible risk of that individual developing rifampicin-resistant TB as the number of *M. tuberculosis* bacilli make it highly unlikely that the relevant mutation were present at the time of SDR administration, and no selective pressure would continue to exist to favour the growth of resistant bacilli. To further minimize the risk, the LPEP protocol excludes from SDR administration all contacts who have taken rifampicin for any reason in the past two years.

### Excluding active TB by screening algorithm

Provider initiated screening for TB, which is done before SDR is given, is best done by symptom screening. Any presumed TB patient is referred to the National TB Programme for further diagnosis.

TB prevalence surveys suggest that 15-38 % of all infectious TB patients show no symptoms, and that 50-75 % of all infectious cases do not fulfil the classic criteria to suspect TB listed above [20, 21]. An evaluation of the screening algorithm for TB showed that the sensitivity of the screening increased substantially when ‘coughing for any duration’ was used for symptom screening, instead of ‘coughing for > 2 weeks’ (from 46.8 % to 59.5 %). Also, TB symptom screening is less sensitive when used for provider-initiated TB screening (i.e. active case finding), and the sensitivity is also negatively affected by the HIV status [22].

The positive and negative predictive value (NPV: the likelihood that a person who tested negative in TB screening does not have culture-positive TB) of a TB screening test is dependent on the prevalence of TB disease in the community screened [23]. In summary, no screening strategy achieves perfect sensitivity and specificity as all strategies will miss some TB patients or declare an unacceptably high proportion of uninfected individuals as TB suspects. Nevertheless, it is universally accepted that TB symptom screening provides a quick, cheap and convenient way to identify individuals with a high risk of TB disease, who then need to undergo further investigation with more definitive tests such as chest radiography, sputum microscopy, Xpert MTB/Rif and culture.

The WHO, with partners, has developed a guideline on screening for active TB [24], based on four systematic reviews and a series of expert consultations [25]. The options for initial screening of adults and children aged 10 years and older include TB symptom screening or screening with chest radiography, which is more sensitive but also more expensive.

## Discussion

The main benefit of the SDR prophylaxis for contacts of leprosy patients is that it reduces their risk of developing leprosy by about 60 %. By giving SDR only to those contacts that do not have any of the classical TB symptoms the likelihood of giving it to a person with TB, especially one with pulmonary cavitary disease, is minimised. Furthermore, a single dose, as opposed to a repeated or a prolonged course of monotherapy, is not expected to have the power to cause selection of resistant mutants even if inadvertently given to contacts with a high bacterial load. None the less, repeated use of rifampicin prophylaxis in an individual, who for example may be a contact of multiple leprosy patients, should be minimised. Repeated use of rifampicin prophylaxis would be expected to increase the risk of selecting resistance slightly but the degree of this risk has not been determined. The quality of the drug should be assured, the logistics of its distribution should be controlled, and the intake of the single dose should be supervised. In areas with a known high prevalence of primary MDR-TB caution is needed because exposure to rifampicin in such areas may provide an advantage for already drug-resistant *M. tuberculosis* [26].

The 60 % reduction in the risk that contacts of leprosy patients will develop leprosy is an important benefit that, according to the opinion of the experts convened in this meeting, far outweighs the theoretical and extremely small chance of selecting rifampicin-resistant *M. tuberculosis*.

Rifampicin prophylaxis also assumes and relies on rifampicin sensitivity of the circulating *Mycobacterium leprae* (*M. leprae*) strains [27]. Regular sampling and molecular monitoring for mutations associated with rifampicin resistance in *M. tuberculosis* as well as in *M. leprae* should be considered in areas where SDR is given to contacts of leprosy patients. Such intensified monitoring of rifampicin resistance in *M. tuberculosis* and *M. leprae* is particularly important in areas with a high rate of primary MDR-TB, and among the recipients of SDR who develop leprosy.

## Conclusion

Based on the proven efficacy in randomized controlled trials, SDR is given to contacts of leprosy patients as PEP. The multi-disciplinary experts in leprosy and TB attending this meeting carefully reviewed and discussed the available evidence regarding the mechanisms and risk factors for the development of (multi) drug-resistance in *M. tuberculosis* with a view to the special situation of the use of SDR as PEP for leprosy. They concluded that SDR given to contacts of leprosy patients, in the absence of symptoms of active TB, poses a negligible risk of generating resistance in *M. tuberculosis* in individuals and at the population level. Thus, the benefits of SDR prophylaxis in reducing the risk of developing leprosy in contacts of new leprosy patients far outweigh the risks of generating drug resistance in *M. tuberculosis*.

### Ethical approval

No ethical approval was required to conduct this meeting.

Participants in the meeting, other than the (co-) authors:

- Dr Indira Padmapani Kahawita, Consultant Dermatologist, Ministry of Health, Colombo, Sri Lanka
- Mr René Stäheli, Director, FAIRMED, Bern, Switzerland

Observer:

- Dr Linh Nguyen, WHO, Global TB Programme, Geneva, Switzerland

## Additional file

Additional file 1: Multilingual abstracts in the six official working languages of the United Nations. (PDF 303 kb)

## Abbreviations

BCG: bacillus calmette-guérin
DNA: deoxyribonucleic acid
EBA: early bactericidal activity
HIV: human immunodeficiency virus
ILEP: international federation of anti-leprosy associations
INH: isoniazid
LPEP: leprosy post-exposure prophylaxis
M: mycobacterium
MDR: multidrug-resistant
NPV: negative predictive value
PEP: post-exposure prophylaxis
SDR: single dose rifampicin
SNPs: single nucleotide polymorphisms
TB: tuberculosis
WHO: World Health Organization

## References

[1] Bakker MI, Hatta M, Kwenang A, Van Benthem BHB, Van Beers SM, Klatser PR (2005). Prevention of leprosy using rifampicin as chemoprophylaxis. Am J Trop Med Hyg.

[2] Moet FJ, Pahan D, Oskam L, Richardus JH, COLEP Study Group (2008). Effectiveness of single dose rifampicin in preventing leprosy in close contacts of patients with newly diagnosed leprosy: cluster randomised controlled trial. BMJ.

[3] Smith CM, Smith WC (2000). Chemoprophylaxis is effective in the prevention of leprosy in endemic countries: a systematic review and meta-analysis. MILEP2 Study Group. Mucosal Immunology of Leprosy. J Infect.

[4] Richardus RA, Alam K, Pahan D (2013). The combined effect of chemoprophylaxis with single dose rifampicin and immunoprophylaxis with BCG to prevent leprosy in contacts of newly diagnosed leprosy cases: a cluster randomized controlled trial (MALTALEP study). BMC Infect Dis.

[5] WHO | Tuberculosis. http://www.who.int/mediacentre/factsheets/fs104/en/. Accessed 17 June 2015.

[6] Gagneux S, DeRiemer K, Van T, Kato-Maeda M, De Jong BC, Narayanan S (2006). Variable host-pathogen compatibility in *Mycobacterium tuberculosis*. Proc Natl Acad Sci U S A.

[7] David HL (1970). Probability distribution of drug-resistant mutants in unselected populations of *Mycobacterium tuberculosis*. Appl Microbiol.

[8] Gagneux S, Long CD, Small PM, Van T, Schoolnik GK, Bohannan BJM (2006). The competitive cost of antibiotic resistance in *Mycobacterium tuberculosis*. Science.

[9] Comas I, Borrell S, Roetzer A, Rose G, Malla B, Kato-Maeda M (2012). Whole-genome sequencing of rifampicin-resistant *Mycobacterium tuberculosis* strains identifies compensatory mutations in RNA polymerase genes. Nat Genet.

[10] Mitchison DA (1984). Drug resistance in Mycobacteria. Br Med Bull.

[11] Colijn C, Cohen T, Ganesh A, Murray M (2011). Spontaneous emergence of multiple drug resistance in tuberculosis before and during therapy. PLoS One.

[12] Den Hertog AL, Menting S, Van Soolingen D, Anthony RM (2014). *Mycobacterium tuberculosis* Beijing genotype resistance to transient rifampin exposure. Emerg Infect Dis.

[13] Chang KC, Leung CC, Grosset J, Yew WW (2011). Treatment of tuberculosis and optimal dosing schedules. Thorax.

[14] Mitchison DA (1998). How drug resistance emerges as a result of poor compliance during short course chemotherapy for tuberculosis. Int J Tuberc Lung Dis Off J Int Union Tuberc Lung Dis.

[15] Jindani A, Aber VR, Edwards EA, Mitchison DA (1980). The early bactericidal activity of drugs in patients with pulmonary tuberculosis. Am Rev Respir Dis.

[16] Selkon JB, Devadatta S, Kulkarni KG, Mitchison DA, Narayana AS, Nair CN (1964). The emergence of isoniazid- resistant cultures in patients with pulmonary tuberculosis during treatment with isoniazid alone or isoniazid plus PAS. Bull World Health Organ.

[17] Mitchison DA, Jindani A, Davies GR, Sirgel F (2007). Isoniazid activity is terminated by bacterial persistence. J Infect Dis.

[18] Vernon A (2013). Treatment of latent tuberculosis infection. Semin Respir Crit Care Med.

[19] Levy SB (2001). Antibiotic resistance: consequences of inaction. Clin Infect Dis Off Publ Infect Dis Soc Am.

[20] 3d APR Union conference 8–11 July 2011, Hong Kong. WHO the STOP TB department. http://www.antitb.org.hk/apr2011/8th%20july/Plenary%20lecture%20I/pl1_Global%20epidemiology%20of%20tuberculosis,%20past,%20present%20and%20future.pdf. Accessed June 2015.

[21] Ayles H, Schaap A, Nota A, Sismanidis C, Tembwe R, De Haas P (2009). Prevalence of tuberculosis, HIV and respiratory symptoms in two Zambian communities: implications for tuberculosis control in the era of HIV. PLoS One.

[22] Corbett EL, Zezai A, Cheung YB, Bandason T, Dauya E, Munyati SS (2010). Provider-initiated symptom screening for tuberculosis in Zimbabwe: diagnostic value and the effect of HIV status. Bull World Health Organ.

[23] Shapiro A, Golub JA. Systematic review of active case-finding strategies in risk groups for tuberculosis (TB) and the relationship to the number needed to screen; report to WHO. 2012. http://www.who.int/tb/Review3NNS_case_active_TB_riskgroups.pdf. Accessed September 2015.

[24] Systematic Screening for Active Tuberculosis: Principles and Recommendations. Geneva: World Health Organization; 2013. http://www.ncbi.nlm.nih.gov/books/NBK294083/. Accessed June 2015. 25996015

[25] Hoog AH van’t, Langendam MW, Mitchell E, Cobelens FG, Sinclair D, Leeflang MMG, et al. A systematic review of the sensitivity and specificity of symptom and chest radiography screening for active pulmonary tuberculosis in HIV negative persons and persons with unknown HIV status. WHO. 2013. http://www.who.int/tb/Review2Accuracyofscreeningtests.pdf?ua=1. Accessed September 2015.

[26] Warren RM, Victor TC, Streicher EM, Richardson M, Beyers N, Van Pittius NC G (2004). Patients with active tuberculosis often have different strains in the same sputum specimen. Am J Respir Crit Care Med.

[27] Cambau E, Chauffour-Nevejans A, Tejmar-Kolar L (2012). Detection of antibiotic resistance in leprosy using GenoType LepraeDR, a novel ready-to-use molecular test. PLoS Negl Trop Dis.

