# Supplementary material for: Negligible risk of inducing resistance in Mycobacterium tuberculosis with single-dose rifampicin as post-exposure prophylaxis for leprosy
Source: Infect Dis Poverty. 2016 Jun 8;5:46. doi: 10.1186/s40249-016-0140-y (PMC4897814; doi:10.1186/s40249-016-0140-y)
Supplement: Additional file 1: — Multilingual abstracts in the six official working languages of the United Nations. (PDF 303 kb) [file 40249_2016_140_MOESM1_ESM.pdf]

Translation of the abstract into the six official working languages of the United Nations

نسبة ضئيلة من مخاطر تحريض المقاومة في المتفطرة السلية مع جرعة ريفامبيسين واحدة كوقاية ما بعد التعرض لمرض الجذام

ليزبيث ميراس، ريتشارد أنطوني، فيم فان براكيل، مارتن و. براتشي، جاك فان دن بروك، ايمانويل كامباو، أريل كافاليرو، كريستا كاسانج، جيثال بيريرا، لي ريتشمان، جان هندريك ريتشاردس، بول سوندرسون، بيتر شتاينمان، وينج واي يو،

الملخص

يُعطى العلاج الوقائي بعد التعرض (PEP) للجذام كجرعة واحدة من ريفامبيسين (SDR) إلى من هم في اتصال مباشر بمرضى الجذام الذين تم تشخيصهم حديثاً. الجرعة الواحدة تقلل من خطر الاصابة بالجذام بين المتصلين بنحو 60٪ في غضون 2-3 سنوات الأولى بعد تلقي الجرعة. في البلدان التي يجري فيها حالياً تنفيذ هذا العلاج الوقائي تحت شروط البرنامج الروتينية في مجالات محددة، أُثيرت أسئلة من قِبَل السلطات الصحية والهيئات المهنية حول المخاطر المحتملة على إحداث مقاومة ريفامبيسين بين سلالات المتفطرة السلية المتداولة في هذه المجالات. لم يتم معالجة هذه المسألة في الأدبيات العلمية حتى الآن. وقد تم عقد اجتماع مع خبراء مرض السل (TB) والجذام، وذلك لتقديم بيان إجماعي موثوق حول مخاطر أن الجرعة الواحدة من ريفا مبيسين من شأنها أن تحفز السل المقاوم للريفامبيسين. وقام الخبراء بمناقشة ومراجعة الأدلة المتوفرة بدقة في ما يتصل بآليات وعوامل الخطر لتطوير (متعدد) مقاومة العقار في المتفطرة السلية بقصد الوضع الخاص لاستخدام الجرعة الواحدة من ريفامبيسين كعلاج وقائي بعد التعرض لمرض الجذام. وخلصوا إلى أن إعطاء جرعة واحدة من ريفامبيسين إلى المتصلين بمرضى الجذام، في حالة عدم وجود أعراض السل النشط، يشكل خطراً لا يذكر في توليد المقاومة للمتفطرة السلية في الأفراد وعلى مستوى السكان. وبالتالي، فإن فوائد العلاج الوقائي لجرعة ريفامبيسين واحدة في الحد من خطر الإصابة بالجذام في المتصلين بمرضى الجذام الجدد يفوق بكثير المخاطر على توليد المقاومة للأدوية في المتفطرة السلية.

Translated from English version into Arabic by Free bird, through

TRANSLATORS WITHOUT BORDERS

单剂量利福平用于麻风病暴露后预防诱导结核杆菌抗性的风险可忽略不计

Liesbeth Mieras, Richard Anthony, Wim van Brakel, Martin W. Bratschi, Jacques van den Broek, Emmanuelle Cambau, Arielle Cavaliero, Christa Kasang, Geethal Perera, Lee Reichman, Jan Hendrik Richardus, Paul Saunderson, Peter Steinmann, Wing Wai Yew

摘要

对于新确诊麻风病患者的接触者可采用单剂量利福平进行暴露后预防。服用单剂量利福平后，接触者前 2-3 年麻风病发病风险降低约 60%。在一些国家，采用单剂量利福平进行麻风病暴露后预防已纳入特定地区的常规防治项目中，但卫生部门和职业机构提出利福平可能导致这些地区结核杆菌抗性诱发的问题。到目前为止，科学文献中尚无关于该问题的定论。为得到一个关于单剂量利福平预防性服药诱导结核病利福平抗性的权威的一致性结论，召开了一个有结核病和麻风病专家参与的会议。专家仔细审阅和讨论了已有的关于结核病（多重）药物抗性发生机制和危险因素的证据，尤其是在单剂量利福平用于麻风病暴露后预防这一特殊背景下。专家最终得出结论：在没有活动性结核病的情况下，可以忽略单剂量利福平用于麻风病接触者在个体和群体水平诱导结合杆菌药物抗性的风险。因此，单剂量利福平预防所能减少的新确诊麻风病患者则的接触者发生麻风病的风险之利大于诱导结核杆菌药物抗性的风险之弊。

Translated from English version into Chinese by Qian Men-Bao, edited by Yang Pin, through

CHINESE CENTER FOR DISEASE CONTROL AND PREVENTION
NATIONAL INSTITUTE OF PARASITIC DISEASES

Risques négligeables de générer une résistance du *Mycobacterium tuberculosis* avec une dose unique de rifampicine comme prophylaxie post-exposition de la lèpre.

Liesbeth Mieras, Richard Anthony, Wim van Brakel, Martin W. Bratschi, Jacques van den Broek, Emmanuelle Cambau, Arielle Cavaliero, Christa Kasang, Geethal Perera, Lee Reichman,
Jan Hendrik Richardus, Paul Saunderson, Peter Steinmann, Wing Wai Yew,

Résumé

La prophylaxie post-exposition (PPE) de la lèpre est administrée en une dose unique de rifampicine (DUR) aux personnes au contact de patients nouvellement diagnostiqués comme étant malades de la lèpre. Avec une DUR, le risque de développer la lèpre chez les personnes en contact avec un malade est réduit d'environ 60% dans les 2 à 3 années après avoir reçu la DUR. Dans les pays où la DUR est actuellement mise en œuvre dans le cadre de conditions d'un programme de routine dans des régions déterminées, les autorités sanitaires et les organismes professionnels ont soulevé des questions sur le risque potentiel de générer une résistance à la rifampicine parmi les souches du *M. tuberculosis* circulant dans ces régions. A ce jour, cette question n'a pas été abordée dans la littérature scientifique. Afin de produire une déclaration officielle de consensus sur les risques que la DUR puisse générer une tuberculose résistante à la rifampicine, une réunion a été organisée avec des spécialistes de la tuberculose (TB) et de la lèpre. Les spécialistes ont soigneusement examiné la question et discuté des preuves disponibles concernant les mécanismes et les facteurs de risques de développement d'une multi-résistance aux médicaments du *M. tuberculosis,* en vue de la situation particulière de l'utilisation de la DUR comme PPE de la lèpre. Ils sont arrivés à la conclusion que la DUR administrée aux personnes en contact avec un malade de la lèpre, en l'absence de symptômes de TB active, ne présente qu'un risque négligeable de générer une résistance du *M. tuberculosis* chez des individus et au niveau de la population. Par conséquent, les avantages de la prophylaxie par la DUR pour réduire le risque de développer la lèpre au contact de patients ayant nouvellement contracté la maladie, dépassent largement les risques de générer une résistance médicamenteuse du *M. tuberculosis*.

Translated from English version into French by Ode Laforge, through

TRANSLATORS
WITHOUT BORDERS

Риск развития лекарственной устойчивости микобактерий туберкулёза *(Mycobacterium tuberculosis)* к рифампицину при однократном приёме препарата в качестве постконтактного профилактического средства у больных лепрой ничтожно мал

Лизабэт Мирас, Ричард Энтони, Вим ван Бракель, Мартин У. Братски, Жак ван ден Брук, Эммануэль Камбей, Ариэль Кавальеро, Криста Касанг, Гитхал Перера, Ли Рейчман, Ян Хендрик Рихардус, Пол Сандерсон, Питер Стейнманн, Винг Вай Ю

Резюме

Постконтактная профилактика (ПКП) лепры (проказы) осуществляется посредством однократного приема препарата рифампицин для пациентов, которым впервые был поставлен диагноз – лепра. Однократный прием препарата рифампицин в первые 2-3 года сокращает риск развития лепры на 60% при контакте с окружающими. В странах, где в настоящее время внедряется однократный прием препарата рифампицин в обычных условиях программы, в определенных районах органами здравоохранения и профессиональными организациями поднимались вопросы о возможном риске развития лекарственно-устойчивых штаммов микобактерий туберкулёза *(M. tuberculosis)* к рифампицину, распространенных в этих

районах. Данный вопрос не рассматривался в научной литературе до настоящего времени. Для разработки официального заключения относительно риска развития лекарственно-устойчивого туберкулеза при однократном приёме препарата было созвано совещание специалистов по туберкулезу и лепре. Эксперты тщательно рассмотрели и обсудили имеющиеся данные относительно механизмов и факторов риска развития устойчивости микобактерий туберкулеза *(M. tuberculosis)* к (разным) лекарственным препаратам с целью использования однократного приема препарата рифампицин в качестве постконтактной профилактики (ПКП) лепры. Они пришли к выводу, что однократный прием препарата рифампицин людьми, контактирующими с больными лепрой, при отсутствии каких-либо симптомов активной формы туберкулеза, представляет ничтожно малый риск развития лекарственной устойчивости микобактерий туберкулёза *(M. tuberculosis)* как для отдельных лиц, так и на уровне населения. Таким образом, преимущества однократного приема препарата рифампицин в качестве профилактического средства для снижения риска развития лепры при контактах с только что заболевшими лепрой значительно перевешивают риски развития лекарственной устойчивости микобактерий туберкулёза *(M. tuberculosis)*.

Translated from English version into Russian by Tatsiana Mankevich, through

TRANSLATORS
WITHOUT BORDERS

Riesgo insignificante de la resistencia inducida en *Mycobacterium tuberculosis* con una dosis única de rifampicina como profilaxis postexposición a la lepra

Liesbeth Mieras, Richard Anthony, Wim van Brakel, Martin W. Bratschi, Jacques van den Broek, Emmanuelle Cambau, Arielle Cavaliero, Christa Kasang, Geethal Perera, Lee Reichman,
Jan Hendrik Richardus, Paul Saunderson, Peter Steinmann, Wing Wai Yew,

Resumen

La profilaxis postexposición a la lepra se administra como una dosis única de rifampicina a los contactos de nuevos pacientes diagnosticados de lepra. La dosis única reduce el riesgo de desarrollo de lepra entre contactos en un 60% aproximadamente durante los 2-3 primeros años tras recibir la dosis única. En los países donde ya se está aplicando la dosis única mediante programas ordinarios en zonas definidas surgieron preguntas por parte de las autoridades sanitarias y las organizaciones profesionales acerca del posible riesgo de inducir la resistencia de la rifampicina entre las cepas de *M. tuberculosis* en estas zonas. Esta cuestión no ha sido tratada hasta ahora en la literatura científica. Con el fin de generar una declaración de consenso sobre el riesgo de que la dosis única provoque una tuberculosis resistente a la rifampicina se convocó una reunión con expertos en tuberculosis y en lepra. Los expertos revisaron minuciosamente y debatieron acerca de las evidencias disponibles acerca de los mecanismos y factores de riesgo para el desarrollo de resistencia a múltiples fármacos en *M. tuberculosis* prestando especial atención a la situación del uso de la dosis única de profilaxis postexposición a la lepra. Su conclusión fue que la dosis única a contactos de pacientes de lepra, en ausencia de síntomas de tuberculosis activa, supone un riesgo insignificante de generar resistencia a *M. tuberculosis* en las personas y a nivel de población. Por tanto, los beneficios que aporta la profilaxis de dos única para reducir el riesgo de desarrollo de lepra en contactos de nuevos pacientes de lepra supera de largo los riesgos de generar resistencia al fármaco en *M. tuberculosis*.

Translated from English version into Spanish by SergioLorenzi, through

TRANSLATORS
WITHOUT BORDERS
